# QSAR Classification Models for Predicting the Activity of Inhibitors of Beta-Secretase (BACE1) Associated with Alzheimer’s Disease

**DOI:** 10.1038/s41598-019-45522-3

**Published:** 2019-06-24

**Authors:** Ignacio Ponzoni, Víctor Sebastián-Pérez, María J. Martínez, Carlos Roca, Carlos De la Cruz Pérez, Fiorella Cravero, Gustavo E. Vazquez, Juan A. Páez, Mónica F. Díaz, Nuria E. Campillo

**Affiliations:** 1 Instituto de Ciencias e Ingeniería de la Computación (UNS-CONICET), Bahía Blanca, Argentina; 2 Departamento de Ciencias e Ingeniería de la Computación, Universidad Nacional del Sur, Bahía Blanca, Argentina; 3 Centro de Investigaciones Biológicas. Consejo Superior de Investigaciones Científicas (CSIC), Ramiro de Maeztu 9, 28040 Madrid, Spain; 4 Planta Piloto de Ingeniería Química – PLAPIQUI (UNS-CONICET), Bahía Blanca, Argentina; 5 Facultad de Ingeniería y Tecnologías, Universidad Católica del Uruguay, Av. 8 de Octubre, 2738 Montevideo, Uruguay; 6 Instituto de Química Médica. Consejo Superior de Investigaciones Científicas (CSIC), Juan de la Cierva 3, 28006 Madrid, Spain; 7 Departamento de Ingeniería Química, Universidad Nacional del Sur (UNS), Bahía Blanca, Argentina

**Keywords:** Virtual drug screening, Medicinal chemistry

## Abstract

Alzheimer’s disease is one of the most common neurodegenerative disorders in elder population. The *β*-site amyloid cleavage enzyme 1 (BACE1) is the major constituent of amyloid plaques and plays a central role in this brain pathogenesis, thus it constitutes an auspicious pharmacological target for its treatment. In this paper, a QSAR model for identification of potential inhibitors of BACE1 protein is designed by using classification methods. For building this model, a database with 215 molecules collected from different sources has been assembled. This dataset contains diverse compounds with different scaffolds and physical-chemical properties, covering a wide chemical space in the drug-like range. The most distinctive aspect of the applied QSAR strategy is the combination of hybridization with backward elimination of models, which contributes to improve the quality of the final QSAR model. Another relevant step is the visual analysis of the molecular descriptors that allows guaranteeing the absence of information redundancy in the model. The QSAR model performances have been assessed by traditional metrics, and the final proposed model has low cardinality, and reaches a high percentage of chemical compounds correctly classified.

## Introduction

Alzheimer’s disease (AD) is a chronic and irreversible brain disorder, which mostly affect to age people. This neurodegenerative disease is characterized by steady cognitive impairment, short-term memory loss, and problems with language. AD constitutes one of the main causes of death in the world, representing the majority of cases of *dementia*^1^. Actually, it is estimated that 47 million people live with dementia around the world, and according to projections it is expected that the number of cases grows to more than 131 million by 2050^2^. Besides, *dementia* has an enormous economic impact. During 2016, the total estimated global cost of *dementia* was US$818 billion, and it will become a trillion-dollar disease by 2018.

Ample experimental studies about the hallmarks of AD conclude that deposition of amyloid plaques in the brains of Alzheimer’s patients constitutes one of the crucial causes of the disease progression^3,4^. The essential component of the amyloid plaques is the amyloid-*β* protein (A*β*)^5^, which is generated by subsequent cleavage of the amyloid precursor protein (APP) by two proteolytic enzymes *β*- and *γ*-secretase^6,7^. The whole biochemical mechanism of proteolytic cleavage depends on the protein-protein interactions between *β*-site amyloid cleavage enzyme 1 (BACE1) and APP^8^. Due to several failures in clinical trials, there is currently some controversy with the use of BACE1 inhibitors. The possible reasons of the failure of BACE1 inhibitors are mainly three. One of them is that BACE1 inhibitors fail as they prevent amyloid production later in the course of illness and may be more effective if used earlier. The second one is about the complexity of AD, given the multifaceted nature of AD, it is unrealistic to expect that BACE1 inhibitors will be work alone^9,10^. And the last reason is the latter reason seems to be related to the side effects, since the conclusions of different studies show that to block completely the activity of BACE1 is not advisable due to severe side effects^11^. But even with these critical points, this way, the inhibition of BACE1, is a promising therapeutic strategy for instance BACE1 inhibitors with a multitarget profile^12^. It is evident that still more effort is needed today in this field.

During last years, several quantitative structure-activity relationships (QSAR) models have been developed in order to predict potential inhibitors for protein BACE1^13–17^. QSAR methods correlate molecular structure to different biological properties such as activity or ADMET properties, providing a relevant data to help during the development of drug design projects^18^. A key step in QSAR studies is the definition or codification of the chemical structure by a diversity of molecular descriptors, such as constitutional, topological, thermodynamic, functional groups, quantum mechanical, geometrical, etc. Nowadays, the development of new cheminformatics software allows to calculate thousands of molecular descriptors^19^, but usually only a small subset of the calculated descriptors brings necessary information for generating the QSAR model of interest. Consequently, the accuracy of these models depends on the correct analysis and selection of computed descriptors as independent variables for the QSAR model definition. For all these reasons, the design of an effective QSAR model constitutes a challenging problem^20^.

Other kind of studies has employed the QSAR models to predict, using a new approach like Fragment Based-QSAR and Group Based-QSAR. This kind of studies consists of counting different chemical fragments and groups in the desired compounds or leads in order to optimize the QSAR models^15,21,22^. Goyal *et al*.^23^ used a group of 20 derivatives of the family of dihydropyridines (DHP) due to their good activities against BACE1 enzyme. Using Vlife MDS software^24^, 705 physicochemical descriptors were obtained and reduced to 311 independent variables. Several selection methods such a step-wise and searching algorithm among others were employed to correlate the biological information to descriptors information. Finally, statistical methods like partial least square (PLS), multiple regression and other techniques were used to obtain models with good correlation and predictive values.

On the other hand, several authors have performed QSAR approaches based on target properties that are correlated to the most important ligand-target interactions^14^ used PoseView^25,26^ and Ligand Explorer software^27^ to find the most important ligand-target interactions of structures deposited in the PDB Bank^28^ visualizing and adjusting the best distance between the atom’s interactions. In this sense, two descriptors were obtained by the last procedure to predict the activity of BACE1 inhibitory compounds, the hydrophobic contacts at 4–5 Å and the number of hydrogen bonds between ligand and target.

Finally, Gupta *et al*.^17^ published a QSAR model integrated by four molecular descriptors that encoded 3D features of the compounds. These descriptors were chosen by combining a ranking approach, which computes the Pearson correlation between each descriptor and the biological activity, and a forward/backward selection approach. Using these descriptors, different QSAR models are inferred by using multiple linear regressions. As a final point, it is interesting to mention that Gupta employs a methodology for computing the molecular descriptors equivalent to the applied in our study, for this reason, we have decided to compare our QSAR models with those obtained by Gupta´s group.

In this paper, we present novel QSAR models for predicting putative inhibitors for protein BACE1. Our proposal combines the application of several machine learning methods, model hybridizing strategies, backward elimination and visual analytics, in order to choose the most informative subset of molecular descriptors for building the QSAR models. Another distinctive aspect of our approach is the characterization of the QSAR modelling problem as a classification system, which helps to achieve a straightforward interpretation of the predictions. The QSAR classification models obtained from our experiments have been analysed, hybridized and compared, from a chemical and mathematical perspective, with Gupta’s models to compare the strengths and weaknesses of proposed models as virtual screening methods.

## Results

### Database

A database with 215 molecules was assembled, where the half maximal inhibitory concentration (IC_50_) values of the compounds were extracted from the literature and web servers (the complete database is shown in the Table S1 of the Supporting Information). All the compounds used on the dataset were obtained from different sources like pharmaceutical industries or web servers like PDB Bank and CheMBL^29^ among others. In order to obtain robust models with a wide applicability domain, the dataset is formed by compounds chemically diverse, with different scaffolds, reflecting a wide chemical space. All of them have been tested experimentally on the BACE1 enzyme with a reported IC_50_ value.

Is important have a representative chemical space in QSAR studies, consequently, is necessary to build a structural diverse set of compounds in order to achieve it. Following this aim, the dataset was analysed characterizing its drug-like properties from a physicochemical point of view. Thus, two different approaches have been used to analyze our datasets diversity, from the point of view of structural and drug-like.

**Figure 1:**
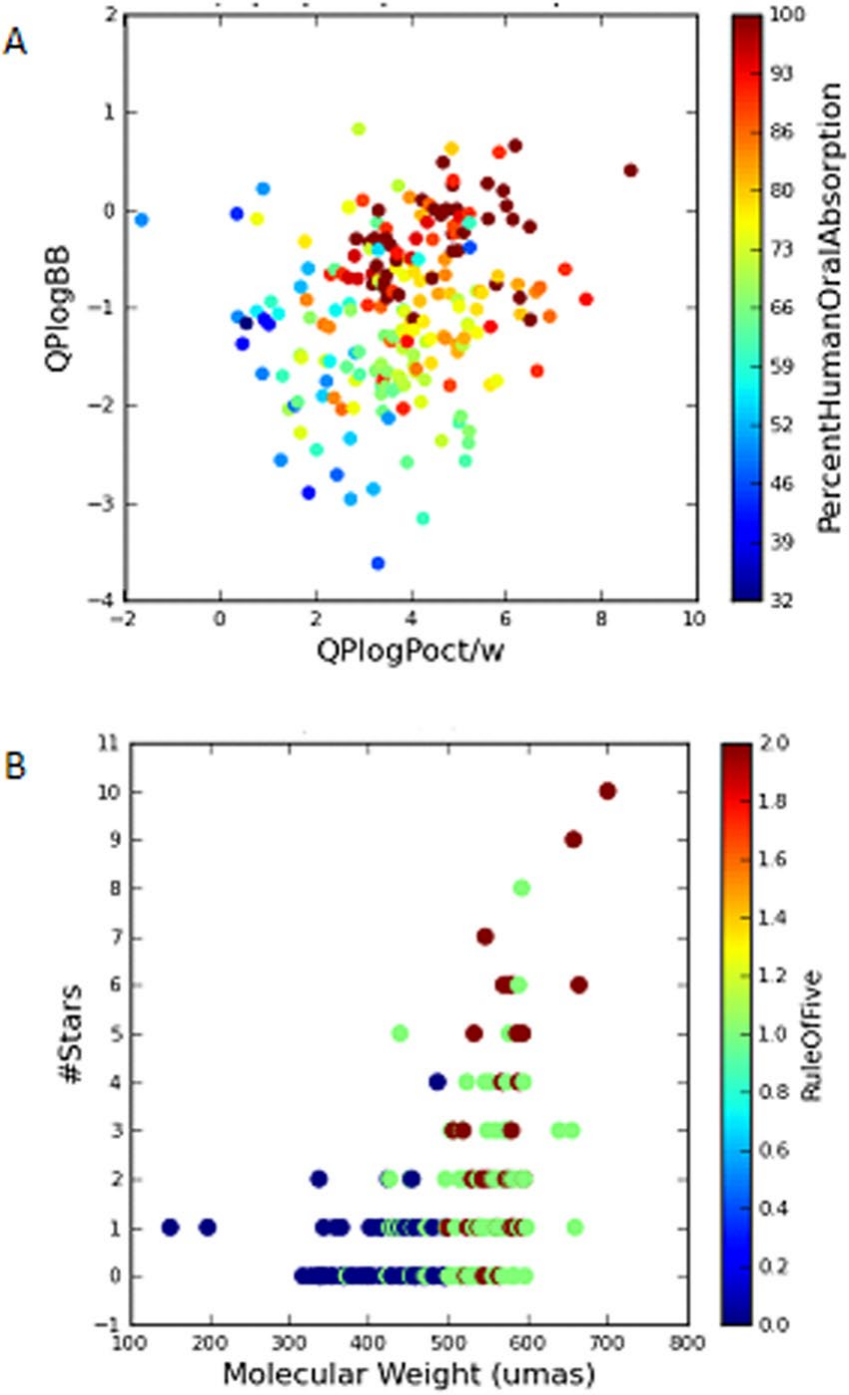
Graphical representation of physicochemical and drug-like properties of the BACE1 dataset. (**A**) Dispersion of compounds regarding logP prediction (x-axis) and logBB prediction (y-axis). Colors are defined by % human oral absorption. (**B**) Dispersion of the dataset according to molecular weight (x-axis) and a parameter related to physical-chemical properties of commercially available drugs (y-axis). The color is defined by the number of violations of the rule of 5.

For the drug-like analysis of the compounds, we used a Qikprop’s module: Small-Molecule Drug Discovery Suite in Schrödinger^30^, which through a set of mathematical functions and structural analysis allows to predict physical-chemical properties such as polar surface area, logP and others. Among the set of properties that the module is able to analyze; for Fig. 1, the molecular weight (MW) was chosen and compared to the coefficient QPlogBB and colored by the percentage of human oral absorption (%); these last two are physical-chemical properties predicted by the program.

Molecular weight property, included as one of the 4 Lipinski’s rules^31^ is one of the parameters analyzed for the dataset. In general, compounds with a molecular weight greater than 500 will not be good candidates as drugs-like compounds, this is considered as a determinant factor. However, in this case, the special topology of the BACE1 enzyme, which presents a large cavity around 10–15 Å, makes important for the inhibition to stablish several protein-ligand interactions in different key points of the pocket far from one another. For this reason, BACE1 drug discovery programs are based in bigger molecules in order to obtain low nanomolar inhibitors comparing with other targets.

The QPlogBB coefficient represents a prediction of the ability of the different compounds to cross the blood-brain barrier for the analysed compounds. Due to the location of the BACE1 enzyme, this property has to be taken into account when selecting compounds that are desired to present some type of inhibitory enzymatic effect *in vivo*. The calculated values of that parameter are in the following interval [−3.0/1.2], the compounds in the dataset present a wide distribution for this value in Fig. 1A. The more positive the value is, the more ability of the compound for crossing the blood-brain barrier. On the contrary, compounds that present negative values theoretically will not be able to cross it satisfactorily. As a result, it is observed that the majority of the compounds in the database is in the upper side of the figure. We can confirm the study with dopamine that present negative values because it is too polar and is not able to cross that barrier well.

Also the (%) Human oral absorption was analyzed due to the fact that drug-like compounds must be absorbed in an adequate rate by the human body as one of the key ADME (Absorption, Distribution, Metabolism and Excretion) properties. This value is obtained through a multiple linear regression of a series of parameters such as the number of rotatable bonds, and the permeability or solubility of the compounds at the time of predicting that property. In the Fig. 1A, we observe that the vast majority of the compounds present values of human oral absorption over 50%.

To conclude the characterization, Fig. 1B illustrates the drug-like behaviour of the dataset. The lower number of starts and violation number of Lipisnki rule, the better drug-like profile. Furthermore, most of these violations are in relation with MW, something expected due to the bigger size of molecules in medicinal chemistry programs for BACE1 inhibitors as stated before. Therefore, as no compounds present more than 2 violations of Lipinski rule of 5, it is possible to confirm that their properties make them likely orally active drugs in humans. A detailed supplementary statistical analysis of several properties is included in the Supporting Information (Table S2 and Figs S1–S6).

### Data pre-processing

As it was mentioned before, the dataset is integrated by 215 molecules. The target variable for this study is BACE1 inhibitory activity (half maximal inhibitory concentration (IC_50_) of each compound. The values are typically expressed as molar concentration. For classification purpose, it was defined one threshold (1000 nM) for discretization of the IC_50_ values in order to define two classes of compounds: High Activity (HA) as inhibitors (IC_50_ ≤ 1000 nM), and Low/Null Activity (LA/NA) as inhibitors (IC_50_ > 1000 nM). Applying this criterion, the 215 molecules of this dataset are distributed in 126 HA compounds and 89 LA compounds.

### Molecular descriptor calculation

DRAGON tool was the software used to compute the Molecular descriptors (MDs)^32^. In Table 1, a summary of the quantity of MDs calculated, clustered by family and type (0D, 1D, 2D, 3D and others), is detailed. A total number of 1867 MDs were finally incorporated to the BACE1 database after removing redundant descriptors with correlation coefficients bigger than 0.95.

**Table 1:** Number of molecular descriptors of each family computed for the database compounds.

Type of Molecular Descriptors	# MD	Type of Molecular Descriptors	# MD
constitutional descriptors	48	geometrical descriptors	74
topological descriptors	119	RDF descriptors	150
walk and path counts	47	3D-MoRSE descriptors	160
connectivity indices	33	WHIM descriptors	99
information indices	47	GETAWAY descriptors	197
2D autocorrelations	96	functional group counts	154
edge adjacency indices	107	atom-centred fragments	120
burden eigenvalues	64	charge descriptors	14
topological charge indices	21	molecular properties	29
eigenvalue-based indices	44	2D binary fingerprints	780
Randic molecular profiles	41	2D frequency fingerprints	780

### *In silico* experimental design

**Figure 2:**
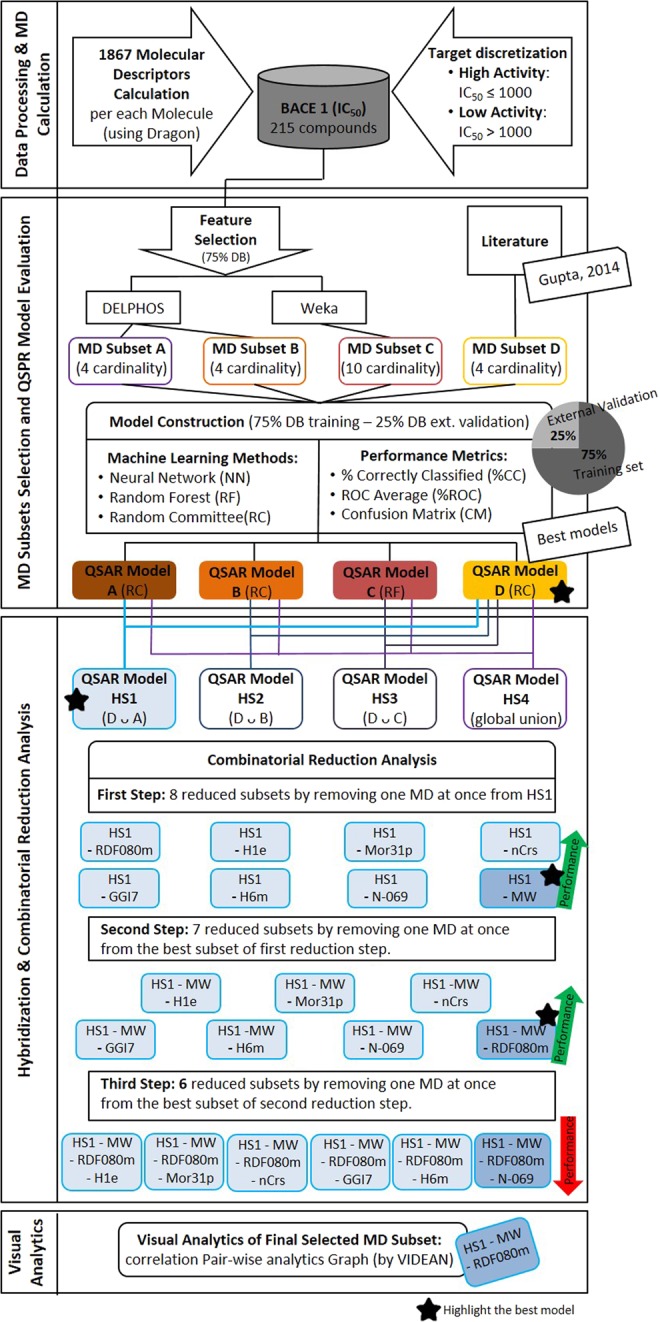
Graphical scheme of experiments reported for the prediction of inhibitors of protein BACE1 by applying QSAR modelling.

The employed protocol to develop QSAR models by feature selection is displayed in Fig. 2. In the first phase the IC_50_ values are discretized using target discretization thresholds explained before. Next, these molecules were optimized to the configuration of minimum energy and, after that, 1867 molecular descriptors were computed using DRAGON software. After that, 25% of the molecules has been left apart for the last step of external validation, and the 75% of the remaining compounds were used for the feature selection and model construction steps. In the second phase, to select the subsets of molecular descriptors (MDs), we used three different approaches from the set of variables returned by DRAGON. The first approach uses DELPHOS tool, which run a machine learning method for selection of MDs in QSAR modelling^33^. DELPHOS infers multiple alternative selections of MDs for defining a QSAR model by applying a wrapper method^34^. In this case, twenty putative subsets had been computed. From them, we chosen two subsets, Subsets A and B (Table 2), since these subsets show the lowest relative absolute error (RAE) values reported by DELPHOS and small numbers of MDs.

**Table 2:** Molecular descriptors of DRAGON associated with the selected subsets.

FS Method	Subset	Cardinality	MDs	Type
DELPHOS	A	4	MW	Constitutional indices
			Mor31p	3D-MoRSE descriptors
			nCrs	Functional group counts
			N-069	Atom-centered fragments
DELPHOS	B	4	MW	Constitutional indices
			piPC04	Walk and path counts
			EEig14d	Eigenvalues
			Mor25p	3D-MoRSE descriptors
WEKA	C	10	nTB	Constitutional indices
			nR03	Ring descriptors
			IC3	Information indices
			G(S.F)	3D Atom Pairs
			nN = C-N<	Functional group counts
			nRNH2	Functional group counts
			C-041	Atom-centered fragments
			B05[C-Cl]	2D Atom Pairs
			F03[C-O]	2D Atom Pairs
			F04[C-C]	2D Atom Pairs
Literature	D	4	H1e	GETAWAY descriptors
			RDF080m	RDF descriptors
			H6m	GETAWAY descriptors
			GGI7	2D autocorrelations

The second one was generated by WEKA tool^35^, applying as feature selection method the Wrapper Subset Evaluator with Random Forest as classifier and Best First technique as Search Method. The selected subset is integrated by ten MDs and it was named Subset C. The most elevated cardinality of this subset is manageable but not desirable, because the physicochemical interpretation of resulting QSAR models usually became a cumbersome and time-consuming process. Besides, the QSAR models integrated by many variables usually suffer of poor generalization in statistical terms. The last one was provided by the scientific literature. In particular, the Subset D corresponds to the selection of four MDs recommended in Gupta *et al*.^17^.

Later, the performance of these four subsets has been evaluated by inferring QSAR classification models. All classifiers have been generated by WEKA software using alternative machine learning methods: the Neural Networks (NN), the Random Forest (RF), and the Random Committee (RC). Recent studies have shown that does not exist a more advisable strategy for learning the QSAR models from the subsets of descriptors^36^. Random Forest and Random Committee are ensemble methods that combine different models with the aim to obtain accurate, robust and stable predictions. The first one implements an ensemble of decision trees where each tree is trained with a random sample of the data and the growth of these trees is carried out with a random selection of features. In a similar way, Random Committee allows building an ensemble of a base classifier that is chosen, for example, a neural network or a decision tree. On the other hand, Neural Networks are configurations of artificial neurons interconnected and organized in different layers to transmit information. The input data crosses the neural network through various operations and then the output values are computed. In this sense, we decided to test these several methods to infer the classifiers. The parameter settings provided by default for WEKA, were used in the experiments for each inference method. Several metrics were calculated using WEKA, regarding the performance assessment: the percentage of cases correctly classified (%CC), the average receiver operating characteristic (ROC) area, and the confusion matrix (CM). In all cases, the stratified sampling and 10-fold cross validation methods provided per default by WEKA were applied. The best QSAR models obtained per each subset is reported in Table 3, where the classifier with best performance is highlighted.

**Table 3:** Performances of the best QSAR classifiers obtained per each subset during external validation. The best model is highlighted in bold.

Subset	Method	%CC	ROC	Confusion Matrix		
A	RC	67	0.71	*High*	*Low*	
				21	10	*High*
				7	14	*Low*
B	RC	69	0.69	*High*	*Low*	
				25	6	*High*
				10	11	*Low*
C	RF	75	0.83	*High*	*Low*	
				26	5	*High*
				8	13	*Low*
***D***	***RC***	***79***	***0*** *.* **8** ***2***	***High***	***Low***	
				***25***	***6***	***High***
				***5***	***16***	***Low***

In the third phase, the first step corresponds to a QSAR model hybridization experiments. These strategies that combine MD subsets obtained from different methodologies has been useful tested in other scenarios^37–41^ and for this reason it was also evaluated in this work. The main goal of these experiments is to improve the accuracy obtained for the best model by adding features included in remaining subsets. Following this idea, three hybridized subsets were defined by combining the subset D, which achieves the best performance in Table 3, with each other subsets in a pairwise fashion. Besides, a fourth hybrid subset was defined as the union of all subsets. These straightforward hybridizations are reported in Table 4.

**Table 4:** Hybridized subset obtained from the union of different subsets.

Hybridized Subset	Combined Subsets	Cardinality
HS1	Subset D ∪ Subset A	8
HS2	Subset D ∪ Subset B	8
HS3	Subset D ∪ Subset C	14
HS4	Union of all subsets	21

Once the hybridized subsets were defined, the next step was the evaluation of them by inferring QSAR classification models employing identical experimental conditions and criteria applied to the original non-hybridized subsets (Table 5). Subset HS1 was the best hybridized subset considering such as accuracy as low cardinality.

**Table 5:** Performances of the best QSAR classifiers obtained per each hybridized subset during external validation. The best model is highlighted in bold.

Subset	Cardinality	Method	%CC	ROC	Confusion Matrix		
***HS1***	***8***	***RC***	**83**	***0.86***	***High***	***Low***	
					**28**	**3**	***High***
					**6**	**15**	***Low***
HS2	8	RF	77	0.79	*High*	*Low*	
					27	4	*High*
					8	13	*Low*
HS3	14	RC	83	0.83	*High*	*Low*	
					28	3	*High*
					6	15	*Low*
HS4	21	RC	85	0.84	*High*	*Low*	
					30	1	*High*
					7	14	*Low*

From this table, it is possible to observe that the QSAR model inferred from the pairwise hybridization between subsets A and D overcame the performance obtained by the models generated from these subsets by alone. However, at this point, it is valid to ask whether there is any subset of the hybridized subset HS1, different from subsets A and D (HS1) that can achieve models with even better performance. This is possible because some of the molecular descriptors provided by subsets A and D can be redundant or introduce some noise to the classification procedure affecting the generalization properties of the QSAR models.

To evaluate the relevance of each molecular descriptor a combinatorial reduction analysis, commonly known as backward elimination was performed. This analysis consists of eliminating a molecular descriptor of HS1 each time and calculating its performance. This process is repeated until not improvement of the performance is observed (see Tables S3–S5 of the Supporting Information). The higher performance was obtained by removing two MD with a cardinality subset of 6. The results of the best QSAR classifiers obtained in each step are included in Table 6, where the minus operator denotes the deletion of one MD from the subset. In the first step, eight subsets with cardinality seven were obtained by removing one molecular descriptor from HS1 at once, and their performances were tested following the same experimental conditions described before. The higher performance is obtained by removing the molecular descriptor MW (%CC = 85%), improving the HS1 accuracy (first row of the Table 6).

**Table 6:** Performances during external validation of the best QSAR classifiers inferred for HS1 reduced subsets in each step. The final model has 6 molecular descriptors, an 85% of cases correctly classified and a ROC curve of 0.88.

Subset	Step	Cardinality	Method	%CC	ROC	Confusion Matrix		
HS1 - MW	1	7	RF	85	0.85	*High*	*Low*	
						28	3	*High*
						5	16	*Low*
***HS1 - MW - RDF080m***	***2***	***6***	***RF***	***85***	***0.88***	***High***	***Low***	
						***30***	***1***	***High***
						***7***	***14***	***Low***
HS1 - MW - –N-069	3	5	RF	83	0.89	*High*	*Low*	
						29	2	*High*
						7	14	*Low*

**Figure 3:**
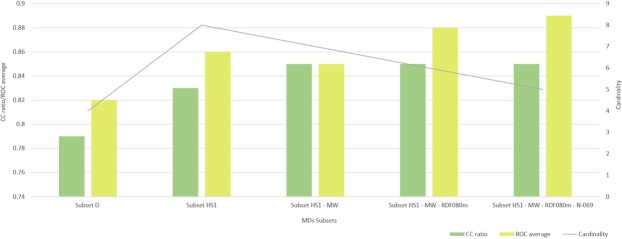
Performance during external validation of the best QSAR model achieved in each experimental step.

In the second reduction step, seven subsets of cardinality six were obtained by alternative removing one MD at once from the reduced subset HS1-MW. The performance of the best QSAR model inferred from each reduced subset is reported in the second row of the Table 6, where the model with higher performance is obtained using HS1-MW-RDF080m. This model preserves the %CC de subset HS1-MW but using one MD less and increases the value of the ROC curve to 0.88. In the third backward elimination step, six subsets of cardinality five were obtained by alternative removing one MD at once from the subset HS1-MW-RDF080m. The performances of the best QSAR model inferred from each of these new reduced subsets are reported in the third row of the Table 6. In this case, the model with higher accuracy corresponds to HS1-MW-RDF080m-N-069, but the performance falls to 83%. For this reason, the final best model (HS1-MW-RDF080m) includes the final selection of MDs is integrated by the following subset of MDs: GGI7, H1e, H6m, Mor31p, N-069 and nCrs. As summary, Fig. 3 shows a performance comparison of best QSAR models obtained in each step of our experimental work. The percentage of corrected classified molecules is expressed as a ratio in order to improve the plot visualization.

It is important to note that this final subset does not fully include the subset A neither the subset D, because one descriptor of both subsets was removed during the reduction of the hybrid subset HS1. One logical question that can be formulated at this point it is why the traditional feature selection approaches (DELPHOS, WEKA and Gupta’s methodology) do not find directly this subset of cardinality six. This occurs because feature selection constitutes a NP-hard problem in computational complexity theory and, therefore, any algorithm used for addressing this problem will explore some part of the huge combinatorial space associated to all possible subsets of MDs. For this reason, the hybridization of subsets obtained by different FS strategies together with the backward elimination can be useful for improving the solutions provided by traditional FS methods.

Concerning to their physicochemical meanings, H1e and H6m are GETAWAY (Geometric Topology and Atom Weights Assembly) descriptors. H1e is an H index autocorrelation of lag 1 weighted by Sanderson electronegativity and H6m is an H index autocorrelation of lag 6 weighted by atomic mass. This type of descriptors is related to the influence of atoms in the determination of the molecular form. On the other hand, GGI7 belong to 2D autocorrelations descriptors class and is the topological charge index of order 7. Another descriptor is nCrs that represents the number of ring secondary C (sp3) and belongs to the Functional group counts. The descriptor N-069 belongs to the Atom-centered fragments class and indicates the number of substructures in which a sp3 nitrogen atom is connected by a simple bond to an electronegative atom or an aromatic substituent. Lastly, Mor31p is a 3D-MoRSe (Molecule Representation of Structures Based on Electron diffraction) descriptor that represents the signal 31 weighted by polarizability. This class of descriptors captures the three-dimensional structure of a molecule and expresses distinctive characteristics of it.

**Figure 4:**
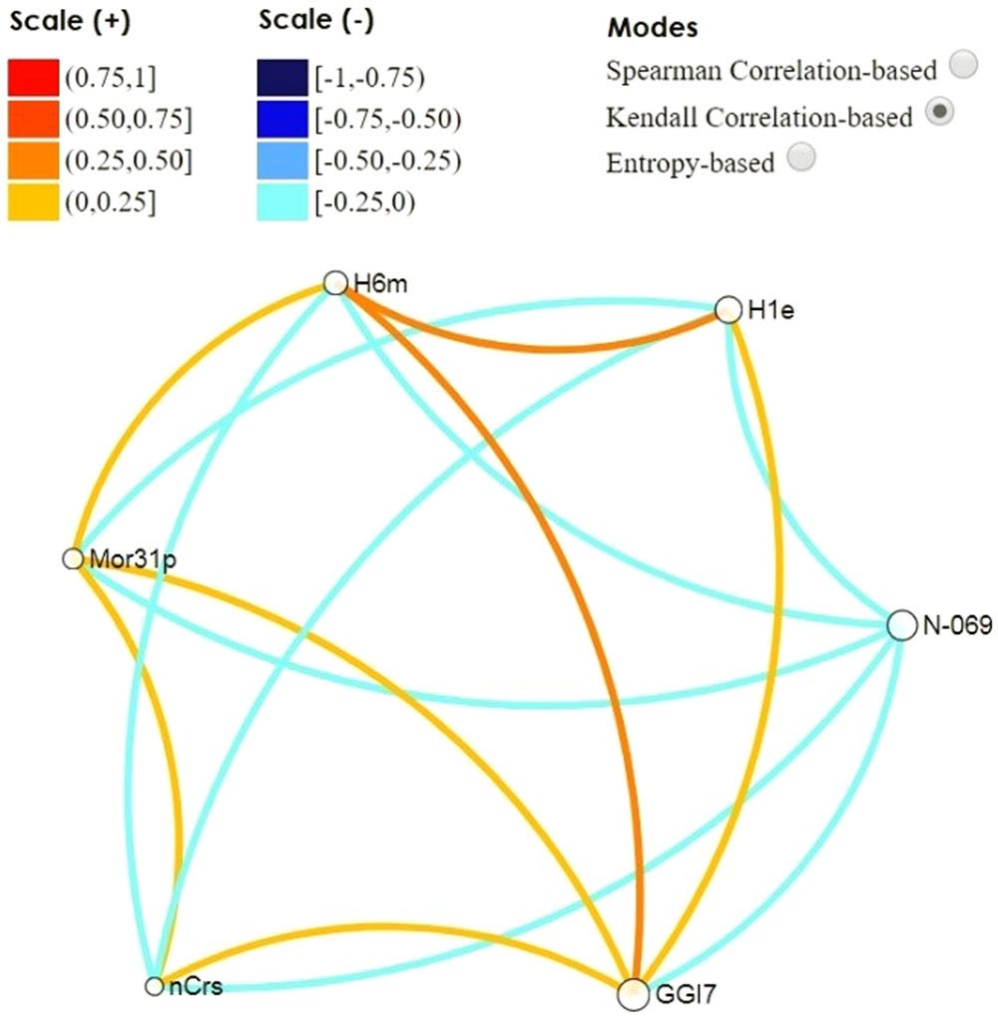
Kendall correlation among descriptors of the best model.

Finally, as last phase of the proposed methodology, it was analyzed the pairs correlation between these six descriptors by using VIDEAN^39^. This tool uses visual analytical methods for molecular descriptors analysis in statistical terms. Figure 4 shows the relationship between the descriptors in the Kendall correlation mode. In this representation, the light orange and light blue tones of the edges (correlation) between the nodes (descriptors) are showing a low level of correlation. This result is the expected one, confirming that each descriptor is contributing unique information to the model.

### Random experiments for assessing the risk of correlation by chance

In this section, we present two random experiments in order to assess the risk of chance correlation both in the final subset of MDs selected by our methodology and in the final QSAR model inferred from these MDs. Whenever in a QSAR modelling method a “best” combination of a few (m) descriptors is selected from a pool of many (M) descriptors in order to best fit given target variable, there is an enhanced risk of chance correlation^42,43^. The risk is boosted (compared to using a predefined subset of descriptors) due to the number of possible models considered, which for increasing m and M speedily exponentially grows to huge orders of magnitude:

$${\rm{MCm}}={\rm{M}}!/({\rm{m}}!(M-{\rm{m}})!)$$

Among so many potential subsets of m descriptors, a few of these combinations are likely to fit the data reasonably well by chance, i.e., without having a true association with the response variable. Therefore, descriptor selection methods, led by a typical measure of fit, are blind with respect to presence or absence of such a true association. Nowadays this problem, also now as selection bias6, has become urgent because the number of molecular descriptors available in computer programs has been significantly increasing during last decades^44^.

In order to address this problem, the first step consists into evaluating if the final subset of MDs selected by our methodology has an accuracy performance significantly higher than the achieved by MD subsets selected randomly. Once this risk can be left behind, the second step should be to determine if our proposed QSAR model, inferred from the final subset of MDs by using machine learning, truly learned how to segregate compounds among the different classes associated to the target variable. In other words, we want to assess if our QSAR model does not classify the compounds randomly.

**Figure 5:**
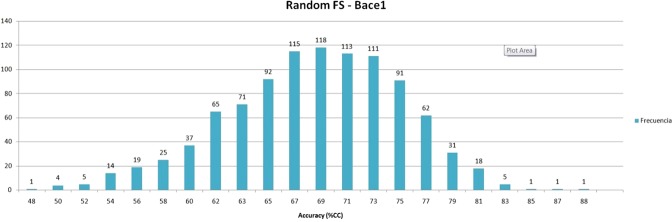
Results of the feature selection randomization experiment.

For the first step, a feature selection randomization experiment (fs-randomization) has been executed. In this experiment, a thousand combinations of six MDs, the same cardinality of the final subset, selected by our methodology, has been randomly selected from the original pool of features integrated by 1867 MDs (see Fig. 5). Therefore, from each random subset, a QSAR model is inferred following the same experimental conditions and criteria used for learning our final QSAR model. Finally, the accuracies (as a percentage of corrected classified samples) of the QSAR models obtained from these random subsets had been computed. The average accuracy (%CC) obtained by the fs-randomization was 69%, and only three of the thousand random trials achieved a performance similar to our final subset (around 85%). Therefore, the performance of the MD subset selected by our methodology is located in the percentile 99 of the accuracies distribution obtained by the fs-randomization, showing a performance significantly higher than the random subsets.

**Figure 6:**
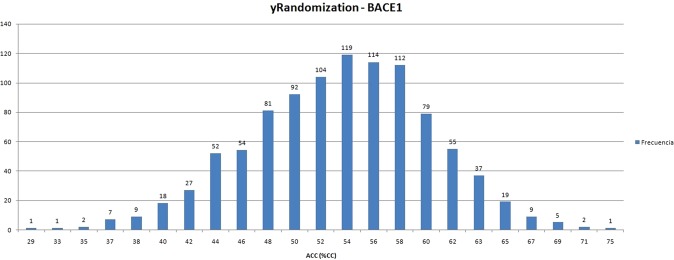
Results of the y-randomization experiment.

For this second step, a y-randomization experiment has been executed. This method consists of randomly shuffled the values of the target variable (y-variable) both in the training set and external validation set, leaving the MDs values intact. Then, a new QSAR model is applied to these scrambled data following the same experimental conditions used to infer the original QSAR model. Every run will yield estimates of the accuracy of the QSAR model, which are recorded. If in each case the scrambled data give much lower accuracy values than the original data, then we can be confident about the relevance of the “real” QSAR model. This methodology is “probably the most powerful validation procedure” for assessing the risk of obtaining QSAR models by chance correlation^45^, and combined with the fs-randomization allows us to achieve confident QSAR models^44^. The average accuracy (%CC) obtained by the y-randomization was 53% (see Fig. 6), and none of the thousand random trials achieved a performance similar to our final subset (best random performance was 75%). Therefore, from these results, we can confidently discard the risk of chance correlation in the final QSAR model proposed in this work.

## Materials and Methods

### Preparation of databases

#### Ligand preparation

The BACE1 dataset on SMILES format were converted to 3D structures using LigPrep^46^ software implemented on Maestro Suite^47^. LigPrep is a 2D-to-3D conversion application that carries out the addition of hydrogen atoms and includes the generation of possible tautomers, stereoisomers and ring conformations using molecular mechanics force fields. The tool also calculates the ionization state of the molecule at a selected pH range. In order to perform our studies, possible states were generated at pH 7.3 with the aim of obtaining the most suitable ionization states of the molecules at physiological pH. The ionization states were assigned with Epik module^48^. Also, no tautomers were generated and all the compounds were desalted. In this process, the search has been restricted to obtain only one low energy ring conformation, as well as one stereoisomer among all that can be found by the tool. The final step of the preparation is an energy minimization of the 3D conformers generated using the OPLS2005 force field^49^.

Different ionization states and conformers of the same molecules were reduced to keep the most suitable 3D structure per initial compound. This preparation is a critical step to carry out further studies with these compounds, such as the physicochemical properties calculation to characterize the dataset.

#### Drug-like properties calculation

All the prepared molecules were studied using Qikprop application of the Small-Molecule Drug Discovery Suite in Schrödinger, an accurate software that predicts structurally significant 2D and 3D properties and pharmaceutically relevant characteristics of chemical compounds. Absorption, Distribution, Metabolism, and Excretion (ADME) properties were predicted using this tool where a total of 44 properties are calculated. The program also calculates properties like molecular weight, polar surface area, molecular volume, QPlogPo/w (predicted octanol/water partition coefficient), number of H-bond donors and acceptor groups, and violations related to the Lipinski’s Rule of 5 and Jorgensen’s Rule of 3 and allows to filter out compounds with clear-cut undesirable properties for drug discovery.

#### Statistical analysis of the database

All the data obtained in the previous step was further analyzed, using SPSS software^50^. Statistical parameters were calculated for the database of compounds, such as modal value, average, median, standard deviation or variance for some key physicochemical parameters of the analysis. Furthermore, several histograms are shown in the Supporting Information where the frequency of the different values can be found grouped by properties.

### Software used for processing molecular descriptors

DRAGON^32^ is a calculation of molecular descriptors tool. It provides almost 5,000 molecular descriptors of different types: 0D, 1D, 2D, and 3D. These molecular descriptors can be used to evaluate molecular structure-activity/property relationships of molecule databases. DRAGON required to calculate their molecular structure files, and also can deal with H-depleted molecules and 2D-structures.

### Machine learning tools used for feature selection and classification models

DELPHOS is a descriptors selection tool that implements a wrapper multi-objective optimization technique based on two phases. In the first phase, a wrapper method and different machine learning algorithms are used to perform an exploration within the search space and find appropriate subsets of descriptors. Then in the second phase, a final subset selection is performed using the selections of the first phase and metrics for a more accurate prediction. In this way, DELPHOS allows identifying relevant subsets of descriptors related to the property under study^33,34,51^.

VIDEAN is a visual analytics tool that combines statistical methods with interactive visualizations for helping in the selection of a subset of molecular descriptors for predicting a target property. It shows the relationships and interactions between the molecular descriptors and the target property in different information panels. A basic visualization used in this work consists in an undirected graph, where nodes represent each descriptor, the size of the node indicates the correlation between this node and the target property, and colored edges reflect the pairwise correlation between the descriptors that it connects. More detailed information about this software is available in Martinez *et al*. 2015^39^.

Weka tool is a machine learning algorithms suit, for data mining tasks^35^. The learning methods used in this work are:

Wrapper Subset Evaluator: method to evaluate a set of attributes using a learning technique. It uses cross validation to estimate the precision^52^.

Best first: is a method that performs searches in the space of subsets of attributes through a greedy hill climbing and using a backtracking strategy.

Neural Networks: a multilayer perceptron method that use back-propagation to classify instances. The network, is allowed to be monitored and modified during training time. The nodes are all sigmoid, except for numeric class.

Random Forest: allows constructing a Random Trees forest^53^. The random trees considers K randomly chosen attributes at each node for build a tree. Performs no pruning, and allow class probabilities estimation (or target mean in the regression case) based on a hold-out set (back fitting).

Random Committee: this method allows to build an ensemble of randomized base classifiers. Each base classifier is built based on the same data, but using a different random number seed. The final prediction is a straight average of all predictions generated by each individual base classifiers.

### Methods used for random experiments

These methods aim to evaluate the risk of random correlation in both the subset of molecular descriptors selected through a specific methodology and in the final QSAR model obtained from these descriptors.

fs-randomization (feature selection randomization): is a method that consists of randomly selecting a number n of molecular descriptors from the original features set, where n is the cardinality of the MDs subset that have been selected by a specific technique. Then, from these random MDs and with the original values of the target property, a new model is inferred following the same experimental criteria that were used to obtain the final QSAR model. Finally, the accuracy value (percentage of correctly classified samples) is reported. This method is applied repeatedly in order to obtain a distribution of values with statistical significance.

y-randomization: is a technique that randomly reorders the values of the target property (y-variable) both in the training set and the external validation set, without modifying the values of the MDs. To apply this technique, the n MDs of the final QSAR model are used. In this way, a new model is generated following the same experimental conditions that were used to obtain the final QSAR model, and the accuracy value is reported. Like fs-randomization, this process is repeated a fixed number of times in order to obtain a distribution of values with statistical significance.

Additional information on these and other techniques for randomizing experiments in QSAR modeling can be found in^44^.

## Conclusions

Alzheimer’s disease is one of the neurodegenerative disorders with stronger impact in elder population around the world. A promising target for its pharmacological treatment is the *β*-site amyloid cleavage enzyme 1 (BACE1). Several studies proposed that BACE1 inhibitors have high therapeutic potential for decelerating the long-term progression of AD, and during last decade several quantitative structure-activity relationships (QSAR) models have been proposed in the literature.

In this paper, a new QSAR model for virtual screening of potential inhibitors of BACE1 protein has been developed using a novel computational strategy. The main goal of the proposed strategy is to obtain accurate QSAR models integrated by a minimum number of molecular descriptors, because models with large number of descriptors suffer of poor generalizability and complex interpretability. QSAR approaches based on machine learning use feature selection methods for choosing the most informative subset of molecular descriptors, but all these algorithms only explore a fraction of the whole combinatorial space of potential subsets. Therefore, these methodologies cannot guarantee a QSAR model of minimum size. For this reason, our proposal combines different methodologies of feature selection, model hybridization approaches, backward elimination and visual analytics to improve the performance of the traditional QSAR methods.

Thanks to this methodology we have developed a robust QSAR model, improving around an 8% regarding the Gupta’s model in terms of both central performance metrics (percentage of corrected classified molecules and average ROC values) by preserving a low cardinality model. Additionally, the risk of chance correlation in the proposed QSAR model has been discarded by executing and analyzing both feature selection randomization and y-randomization experiments. Furthermore, the wide chemical diversity of the database used in our study compared to previous studies enhances the applicability of our model. Therefore, the results obtained by this novel strategy show that our approach contributed to achieve a QSAR model that can be a useful virtual screening method for prediction of BACE1 inhibitors. Nevertheless, as any QSAR model generated by machine learning, it is important to know that these classifiers preserve their levels of accuracy for molecules structurally similar to the chemical compounds used during the training of the model. For this reason, potential users of these models should be employ applicability domain methods. As a future work, we plan to evaluate this new methodology in the development of other classification models and continue testing our QSAR model for predicting BACE1 over other datasets.

### Description of additional data files

The following additional data are available with the online version of this paper. “Supplementary Information” contains more information about the dataset, its statistical properties and additional results about intermediate calculations during the backward elimination analysis.

## Supplementary information

Supplementary Information

## Data Availability

All data generated or analyzed are available upon request.

## References

[1] Burns A, Iliffe S (2009). Alzheimer’s disease. BMJ..

[2] Prince, M., Comas-Herreras, A., Knapp, M., Guerchet, M. & Karagiannidou, M. World Alzheimer report 2016: improving healthcare for people living with dementia: coverage, quality and costs now and in the future (2016).

[3] Guo T, Hobbs DW (2006). Development of BACE1 inhibitors for Alzheimer’s disease. Curr. Med. Chem..

[4] Cole SL, Vassar R (2008). BACE1 structure and function in health and Alzheimer’s disease. Curr. Alzheimer Res..

[5] Selkoe DJ (1999). Translating cell biology into therapeutic advances in Alzheimer’s disease. Nature..

[6] Citron M (2010). Alzheimer’s disease: strategies for disease modification. Nat. Rev. Drug Discovery..

[7] De Strooper B, Vassar R, Golde T (2010). The secretases: enzymes with therapeutic potential in Alzheimer disease. Nat. Rev. Neurol..

[8] Zou L, Yang R, Zhang P, Dai Y (2010). The enhancement of amyloid precursor protein and beta-site amyloid cleavage enzyme 1 interaction: amyloid-beta production with aging. Int. J. Mol. Med..

[9] Coimbra, J. R. *et al*. Highlights in BACE1 inhibitors for Alzheimer’s disease treatment. *Front. Chem*. **6** (2018). 10.3389/fchem.2018.00178 PMC5977085 29881722

[10] Voytyuk I, De Strooper B, Chavez-Gutierrez L (2018). Modulation of γ-and β-secretases as early prevention against Alzheimer’s disease. Biol. Psychiatry..

[11] Chatila Zena K., Kim Eunhee, Berlé Clara, Bylykbashi Enjana, Rompala Alexander, Oram Mary K., Gupta Drew, Kwak Sang Su, Kim Young Hye, Kim Doo Yeon, Choi Se Hoon, Tanzi Rudolph E. (2018). BACE1 Regulates Proliferation and Neuronal Differentiation of Newborn Cells in the Adult Hippocampus in Mice. eneuro.

[12] González-Naranjo P (2019). Indazolylketones as new multitarget cannabinoid drugs. Eur. J. Med. Chem..

[13] Manoharan P, Vijayan RS, Ghoshal N (2010). Rationalizing fragment based drug discovery for BACE1: insights from FB-QSAR, FB-QSSR, multi objective (MO-QSPR) and MIF. studies. J. Comput.-Aided Mol. Des..

[14] Nastase AF, Boyd DB (2012). Simple structure-based approach for predicting the activity of inhibitors of beta-secretase (BACE1) associated with Alzheimer’s disease. J. Chem. Inf. Model..

[15] Huang D (2013). Comprehensive 3D-QSAR and binding mode of BACE-1 inhibitors using R-group search and molecular docking. J. Mol. Graphics Modell..

[16] Chakraborty S, Ramachandran B, Basu S (2014). Encompassing receptor flexibility in virtual screening using ensemble docking-based hybrid QSAR: discovery of novel phytochemicals for BACE1 inhibition. Mol. BioSyst..

[17] Gupta K (2014). Qsar studies on gallic acid derivatives and molecular docking studies of Bace1. enzyme–A potent target of Alzheimer disease. BIOEJ..

[18] Sullivan KM, Manuppello JR, Willett CE (2014). Building on a solid foundation: SAR and QSAR as a fundamental strategy to reduce animal testing. SAR QSAR Environ. Res..

[19] Khan AU (2016). Descriptors and their selection methods in QSAR analysis: paradigm for drug design. Drug discovery today..

[20] Shahlaei M (2013). Descriptor selection methods in quantitative structure-activity relationship studies: a review study. Chem. Rev...

[21] Klebe G, Abraham U, Mietzner T (1994). Molecular similarity indices in a comparative analysis (CoMSIA) of drug molecules to correlate and predict their biological activity. J. Med. Chem..

[22] Pandey A, Mungalpara J, Mohan CG (2010). Comparative molecular field analysis and comparative molecular similarity indices analysis of hydroxyethylamine derivatives as selective human BACE-1 inhibitor. Mol. Diversity..

[23] Goyal S, Dhanjal JK, Tyagi C, Goyal M, Grover A (2014). Novel fragment-based QSAR modeling and combinatorial design of pyrazole-derived CRK3 inhibitors as potent antileishmanials. Chem. Biol. Drug Des..

[24] VLifeMDS: Molecular Design Suite, Pune, India, 3rd edition (2004).

[25] Stierand K, Rarey M (2011). Consistent two-dimensional visualization of protein-ligand complex series. J. Cheminf..

[26] Schomburg K, Ehrlich HC, Stierand K, Rarey M (2010). From structure diagrams to visual chemical patterns. J. Chem. Inf. Model..

[27] Moreland JL, Gramada A, Buzko OV, Zhang Q, Bourne PE (2005). The Molecular Biology Toolkit (MBT): a modular platform for developing molecular visualization applications. BMC Bioinf..

[28] Berman HM (2000). The Protein Data Bank. Nucleic Acids Res..

[29] Gaulton A (2012). ChEMBL: a large-scale bioactivity database for drug discovery. Nucleic Acids Res..

[30] QikProp, v. S., Schrödinger (2015).

[31] Lipinski CA, Lombardo F, Dominy BW, Feeney PJ (2001). Experimental and computational approaches to estimate solubility and permeability in drug discovery and development settings. Adv. Drug Delivery Rev..

[32] Dragon, Version **5.5**, Talete srl (2007).

[33] Soto, A. J., Martínez, M. J., Cecchini, R. L., Vazquez, G. E. & Ponzoni, I. DELPHOS: computational tool for selection of relevant descriptor subsets in ADMET prediction. *1st International Meeting of Pharmaceutical Sciences*. (2010).

[34] Soto AJ, Cecchini RL, Vazquez GE, Ponzoni I (2009). Multi‐objective feature selection in QSAR using a machine learning approach. QSAR Comb. Sci..

[35] Eibe, F., Hall, M. A. & Witten, I. H. The WEKA workbench. Online appendix for “Data Mining: practical machine learning tools and techniques”. (Morgan Kaufmann, 2016).

[36] Eklund M, Norinder U, Boyer S, Carlsson L (2014). Choosing feature selection and learning algorithms in QSAR. J. Chem. Inf. Model...

[37] Mansouri K, Ringsted T, Ballabio D, Todeschini R, Consonni V (2013). Quantitative structure-activity relationship models for ready biodegradability of chemicals. J. Chem. Inf. Model..

[38] Zakharov AV, Peach ML, Sitzmann M, Nicklaus MC (2014). QSAR modeling of imbalanced high-throughput screening data in PubChem. J. Chem. Inf. Model..

[39] Martinez MJ, Ponzoni I, Diaz MF, Vazquez GE, Soto AJ (2015). Visual analytics in cheminformatics: user-supervised descriptor selection for QSAR methods. J. Cheminf..

[40] Ponzoni I (2017). Hybridizing Feature Selection and Feature Learning Approaches in QSAR Modeling for Drug Discovery. Sci. Rep..

[41] Cravero F, Martinez MJ, Vazquez GE, Diaz MF, Ponzoni I (2016). Feature learning applied to the estimation of tensile strength at break in polymeric material design. J. Integr. Bioinform..

[42] Topliss JG, Costello RJ (1972). Change correlations in structure-activity studies using multiple regression analysis. J. Med. Chem..

[43] Topliss JG, Edwards RP (1979). Chance factors in studies of quantitative structure-activity relationships. J. Med. Chem..

[44] Rucker C, Rucker G, Meringer M (2007). y-Randomization and its variants in QSPR/QSAR. J. Chem. Inf. Model..

[45] Kubinyi, H. QSAR in Drug Design in *Handbook of* Chemoinformatics, (ed. Gasteiger, J.) 1532-1554 (Wiley-VCH, 2003).

[46] LigPrep, version **3.1**, Schrödinger (2015).

[47] Maestro, version **9.9**, Schrödinger (2014).

[48] Epik, version **3.1**, Schrödinger (2015).

[49] Jorgensen WL, Tirado-Rives J (1988). The OPLS [optimized potentials for liquid simulations] potential functions for proteins, energy minimizations for crystals of cyclic peptides and crambin. J. Am. Chem. Soc..

[50] IBM SPSS. Statistics for Windows, Version **22.0**, IBM Corp (2013).

[51] Soto, A. J., Cecchini., R. L., Vazquez, G. E. & Ponzoni, I. A wrapper-based feature selection method for ADMET prediction using evolutionary computing. *Evolutionary Computation, Machine Learning and Data Mining in Bioinformatics*. 188–189 (2008).

[52] Kohavi R, John GH (1997). Wrappers for feature subset selection. Artif. Intell...

[53] Breiman L (2001). Random Forest. Mach. Learn..

