# Supplementary material for: QSAR Classification Models for Predicting the Activity of Inhibitors of Beta-Secretase (BACE1) Associated with Alzheimer’s Disease
Source: Sci Rep. 2019 Jun 24;9:9102. doi: 10.1038/s41598-019-45522-3 (PMC6591229; doi:10.1038/s41598-019-45522-3)
Supplement: Supplementary file 1 — Supplementary Information [file 41598_2019_45522_MOESM1_ESM.doc]

**QSAR Classification Models for Predicting the Activity of Inhibitors of Beta-Secretase (BACE1) Associated with Alzheimer’s Disease**

Ignacio Ponzoni^1,2, §, *^, Víctor Sebastián-Pérez^3, §^, María J. Martínez^1,2, §^, Carlos Roca^3^, Carlos De la Cruz Pérez^3^, Fiorella Cravero^4^, Gustavo E. Vazquez^5^, Juan A. Páez^6^, Mónica F. Díaz^4,7^ and Nuria E. Campillo^3,*^

^1^ Instituto de Ciencias e Ingeniería de la Computación (UNS-CONICET), Bahía Blanca, Argentina.

^2^ Departamento de Ciencias e Ingeniería de la Computación, Universidad Nacional del Sur, Bahía Blanca, Argentina.

^3^ Centro de Investigaciones Biológicas. Consejo Superior de Investigaciones Científicas (CSIC), Ramiro de Maeztu 9, 28040 Madrid, Spain.

^4^ Planta Piloto de Ingeniería Química – PLAPIQUI (Universidad Nacional del Sur-CONICET) Bahía Blanca, Argentina.

^5^ Facultad de Ingeniería y Tecnologías, Universidad Católica del Uruguay. Av. 8 de Octubre 2738, Montevideo, Uruguay.

^6^ Instituto de Química Médica. Consejo Superior de Investigaciones Científicas (CSIC), Juan de la Cierva 3, 28006 Madrid, Spain.

^7^ Departamento de Ingeniería Química, Universidad Nacional del Sur (UNS), Bahía Blanca, Argentina.

^§^ These authors equally contributed to this work.

*corresponding authors: [ip@cs.uns.edu.ar](mailto:ip@cs.uns.edu.ar); [nuria.campillo@csic.es](mailto:nuria.campillo@csic.es);

**Table S1 Compounds of the training set for predictive models**

| **Number of compound** | **Ligand ID** | **IC50 (nM)** | **Obtained from** | **Activity class** |
| --- | --- | --- | --- | --- |
| **Compound 1** | 133793 | 0.037 | Actives Combined DUD-E | High Activity |
| **Compound 2** | 352385 | 0,078 | Actives Combined DUD-E | High Activity |
| **Compound 3** | ZPQ | 0,3 | PDB bank | High Activity |
| **Compound 4** | 413436 | 1 | Actives Combined DUD-E | High Activity |
| **Compound 5** | 23I | 1,1 | PDB bank | High Activity |
| **Compound 6** | GRL8234 | 1,8 | NCBI | High Activity |
| **Compound 7** | PB0 | 2 | PDB bank | High Activity |
| **Compound 8** | 384447 | 2 | Actives Combined DUD-E | High Activity |
| **Compound 9** | 384953 | 2 | Actives Combined DUD-E | High Activity |
| **Compound 10** | BJC | 2,5 | PDB bank | High Activity |
| **Compound 11** | PB7 | 5 | PDB bank | High Activity |
| **Compound 12** | E2609 | 7 | NCBI | High Activity |
| **Compound 13** | CHEMBL566603 | 8,8 | Actives ChEMBl | High Activity |
| **Compound 14** | 509542 | 9 | Actives Combined DUD-E | High Activity |
| **Compound 15** | 591101 | 9 | Actives Combined DUD-E | High Activity |
| **Compound 16** | PB8 | 9,5 | PDB bank | High Activity |
| **Compound 17** | 413448 | 10 | Actives Combined DUD-E | High Activity |
| **Compound 18** | 423771 | 10 | Actives Combined DUD-E | High Activity |
| **Compound 19** | 509618 | 10 | Actives Combined DUD-E | High Activity |
| **Compound 20** | 509471 | 10 | Actives Combined DUD-E | High Activity |
| **Compound 21** | 566548 | 10 | Actives Combined DUD-E | High Activity |
| **Compound 22** | 351667 | 10 | Actives Combined DUD-E | High Activity |
| **Compound 23** | 0V6 | 11 | PDB bank | High Activity |
| **Compound 24** | CHEMBL378225 | 15 | Actives ChEMBl | High Activity |
| **Compound 25** | 10O | 18 | PDB bank | High Activity |
| **Compound 26** | 3RS | 18 | PDB bank | High Activity |
| **Compound 27** | 522287 | 19 | Actives Combined DUD-E | High Activity |
| **Compound 28** | Z81 | 20 | PDB bank | High Activity |
| **Compound 29** | LY2886721 | 20,3 | NCBI | High Activity |
| **Compound 30** | 413493 | 22 | Actives Combined DUD-E | High Activity |
| **Compound 31** | 373508 | 23 | Actives Combined DUD-E | High Activity |
| **Compound 32** | 415395 | 24 | Actives Combined DUD-E | High Activity |
| **Compound 33** | 591027 | 27 | Actives Combined DUD-E | High Activity |
| **Compound 34** | ZOO | 32 | PDB bank | High Activity |
| **Compound 35** | 581091 | 33 | Actives Combined DUD-E | High Activity |
| **Compound 36** | 413478 | 38 | Actives Combined DUD-E | High Activity |
| **Compound 37** | 581129 | 38 | Actives Combined DUD-E | High Activity |
| **Compound 38** | 436812 | 39 | Actives Combined DUD-E | High Activity |
| **Compound 39** | 1M7 | 40 | PDB bank | High Activity |
| **Compound 40** | 422018 | 40 | Actives Combined DUD-E | High Activity |
| **Compound 41** | 423774 | 40 | Actives Combined DUD-E | High Activity |
| **Compound 42** | 10Q | 48 | PDB bank | High Activity |
| **Compound 43** | 3UW | 48 | PDB bank | High Activity |
| **Compound 44** | CHEMBL370043 | 57 | Actives ChEMBl | High Activity |
| **Compound 45** | 842 | 59 | PDB bank | High Activity |
| **Compound 46** | 1B1 | 79 | PDB bank | High Activity |
| **Compound 47** | CS5 | 80 | PDB bank | High Activity |
| **Compound 48** | 574537 | 80 | Actives Combined DUD-E | High Activity |
| **Compound 49** | 591032 | 83 | Actives Combined DUD-E | High Activity |
| **Compound 50** | 1YU | 88 | PDB bank | High Activity |
| **Compound 51** | 554872 | 89 | Actives Combined DUD-E | High Activity |
| **Compound 52** | CHEMBL560497 | 91 | Actives ChEMBl | High Activity |
| **Compound 53** | 3BN | 98 | PDB bank | High Activity |
| **Compound 54** | 337554 | 98 | Actives Combined DUD-E | High Activity |
| **Compound 55** | 438409 | 99 | Actives Combined DUD-E | High Activity |
| **Compound 56** | 384450 | 100 | Actives Combined DUD-E | High Activity |
| **Compound 57** | 384448 | 100 | Actives Combined DUD-E | High Activity |
| **Compound 58** | 422050 | 100 | Actives Combined DUD-E | High Activity |
| **Compound 59** | 438134 | 100 | Actives Combined DUD-E | High Activity |
| **Compound 60** | 581092 | 100 | Actives Combined DUD-E | High Activity |
| **Compound 61** | 683702 | 100 | Actives Combined DUD-E | High Activity |
| **Compound 62** | 353482 | 100 | Actives Combined DUD-E | High Activity |
| **Compound 63** | 574604 | 100 | Actives Combined DUD-E | High Activity |
| **Compound 64** | 419741 | 130 | Actives Combined DUD-E | High Activity |
| **Compound 65** | 574615 | 130 | Actives Combined DUD-E | High Activity |
| **Compound 66** | 583420 | 130 | Actives Combined DUD-E | High Activity |
| **Compound 67** | 364435 | 138 | Actives Combined DUD-E | High Activity |
| **Compound 68** | JNJ715754 | 140 | Janssen | High Activity |
| **Compound 69** | CHEMBL237493 | 140 | Actives ChEMBl | High Activity |
| **Compound 70** | 509388 | 140 | Actives Combined DUD-E | High Activity |
| **Compound 71** | 486415 | 140 | Actives Combined DUD-E | High Activity |
| **Compound 72** | 581094 | 140 | Actives Combined DUD-E | High Activity |
| **Compound 73** | 601115 | 140 | Actives Combined DUD-E | High Activity |
| **Compound 74** | 437805 | 142 | Actives Combined DUD-E | High Activity |
| **Compound 75** | 438408 | 149 | Actives Combined DUD-E | High Activity |
| **Compound 76** | 0KQ | 150 | PDB bank | High Activity |
| **Compound 77** | 457257 | 150 | Actives Combined DUD-E | High Activity |
| **Compound 78** | 712 | 163 | PDB bank | High Activity |
| **Compound 79** | MK8931 | 239 | NCBI | High Activity |
| **Compound 80** | X17 | 270 | PDB bank | High Activity |
| **Compound 81** | 437844 | 303 | Actives Combined DUD-E | High Activity |
| **Compound 82** | 419735 | 314 | Actives Combined DUD-E | High Activity |
| **Compound 83** | 581123 | 330 | Actives Combined DUD-E | High Activity |
| **Compound 84** | 437941 | 350 | Actives Combined DUD-E | High Activity |
| **Compound 85** | 575535 | 350 | Actives Combined DUD-E | High Activity |
| **Compound 86** | 581082 | 350 | Actives Combined DUD-E | High Activity |
| **Compound 87** | 582097 | 350 | Actives Combined DUD-E | High Activity |
| **Compound 88** | 682703 | 350 | Actives Combined DUD-E | High Activity |
| **Compound 89** | 384460 | 360 | Actives Combined DUD-E | High Activity |
| **Compound 90** | 566508 | 360 | Actives Combined DUD-E | High Activity |
| **Compound 91** | 437685 | 365 | Actives Combined DUD-E | High Activity |
| **Compound 92** | 437888 | 365 | Actives Combined DUD-E | High Activity |
| **Compound 93** | 1BE | 400 | PDB bank | High Activity |
| **Compound 94** | 3KT | 430 | PDB bank | High Activity |
| **Compound 95** | 1H6 | 435 | PDB bank | High Activity |
| **Compound 96** | 684246 | 440 | Actives Combined DUD-E | High Activity |
| **Compound 97** | 337707 | 450 | Actives Combined DUD-E | High Activity |
| **Compound 98** | 683699 | 460 | Actives Combined DUD-E | High Activity |
| **Compound 99** | 620864 | 460 | Actives Combined DUD-E | High Activity |
| **Compound 100** | 423973 | 500 | Actives Combined DUD-E | High Activity |
| **Compound 101** | 366487 | 500 | Actives Combined DUD-E | High Activity |
| **Compound 102** | 419724 | 500 | Actives Combined DUD-E | High Activity |
| **Compound 103** | 353633 | 510 | Actives Combined DUD-E | High Activity |
| **Compound 104** | 583479 | 520 | Actives Combined DUD-E | High Activity |
| **Compound 105** | 556161 | 550 | Actives Combined DUD-E | High Activity |
| **Compound 106** | 683087 | 560 | Actives Combined DUD-E | High Activity |
| **Compound 107** | 437718 | 570 | Actives Combined DUD-E | High Activity |
| **Compound 108** | RG7129 | 570 | Roche | High Activity |
| **Compound 109** | 646031 | 580 | Actives Combined DUD-E | High Activity |
| **Compound 110** | 683159 | 580 | Actives Combined DUD-E | High Activity |
| **Compound 111** | 615575 | 580 | Actives Combined DUD-E | High Activity |
| **Compound 112** | CHEMBL391087 | 590 | Actives ChEMBl | High Activity |
| **Compound 113** | 400989 | 590 | Actives Combined DUD-E | High Activity |
| **Compound 114** | AZD3293 | 600 | Astra Zeneca | High Activity |
| **Compound 115** | CHEMBL1208873 | 810 | Actives ChEMBl | High Activity |
| **Compound 116** | 620781 | 810 | Actives Combined DUD-E | High Activity |
| **Compound 117** | 472950 | 811 | Actives Combined DUD-E | High Activity |
| **Compound 118** | 682907 | 820 | Actives Combined DUD-E | High Activity |
| **Compound 119** | 616133 | 822 | Actives Combined DUD-E | High Activity |
| **Compound 120** | 438027 | 850 | Actives Combined DUD-E | High Activity |
| **Compound 121** | 620778 | 850 | Actives Combined DUD-E | High Activity |
| **Compound 122** | 436764 | 860 | Actives Combined DUD-E | High Activity |
| **Compound 123** | 591089 | 860 | Actives Combined DUD-E | High Activity |
| **Compound 124** | XX4 | 900 | PDB bank | High Activity |
| **Compound 125** | CHEMBL485319 | 905 | Actives ChEMBl | High Activity |
| **Compound 126** | CHEMBL583888 | 950 | Actives ChEMBl | High Activity |
| **Compound 127** | 0GH | 1000 | PDB bank | Low Activity |
| **Compound 128** | 344718 | 1000 | Inactives DUD-E | Low Activity |
| **Compound 129** | PGN174 | 1000 | CIB | Low Activity |
| **Compound 130** | PGN170 | 1000 | CIB | Low Activity |
| **Compound 131** | LPD | 1000 | PDB | Low Activity |
| **Compound 132** | 0GU | 3530 | PDB bank | Low Activity |
| **Compound 133** | L655240 | 4470 | NCBI | Low Activity |
| **Compound 134** | 344463 | 5000 | Inactives DUD-E | Low Activity |
| **Compound 135** | 345063 | 5000 | Inactives DUD-E | Low Activity |
| **Compound 136** | 415208 | 6700 | Inactives DUD-E | Low Activity |
| **Compound 137** | 415390 | 6700 | Actives Combined DUD-E | High Activity |
| **Compound 138** | 09B | 7000 | PDB bank | Low Activity |
| **Compound 139** | 54M | 7900 | PDB bank | Low Activity |
| **Compound 140** | 349889 | 10000 | Inactives DUD-E | Low Activity |
| **Compound 141** | 350006 | 10000 | Inactives DUD-E | Low Activity |
| **Compound 142** | 350007 | 10000 | Inactives DUD-E | Low Activity |
| **Compound 143** | 350034 | 10000 | Inactives DUD-E | Low Activity |
| **Compound 144** | 352422 | 10000 | Inactives DUD-E | Low Activity |
| **Compound 145** | 352714 | 10000 | Inactives DUD-E | Low Activity |
| **Compound 146** | 352775 | 10000 | Inactives DUD-E | Low Activity |
| **Compound 147** | 352852 | 10000 | Inactives DUD-E | Low Activity |
| **Compound 148** | 352909 | 10000 | Inactives DUD-E | Low Activity |
| **Compound 149** | 352951 | 10000 | Inactives DUD-E | Low Activity |
| **Compound 150** | 352952 | 10000 | Inactives DUD-E | Low Activity |
| **Compound 151** | 353281 | 10000 | Inactives DUD-E | Low Activity |
| **Compound 152** | 415392 | 10000 | Inactives DUD-E | Low Activity |
| **Compound 153** | 353448 | 10000 | Inactives DUD-E | Low Activity |
| **Compound 154** | 353537 | 10000 | Inactives DUD-E | Low Activity |
| **Compound 155** | 353586 | 10000 | Inactives DUD-E | Low Activity |
| **Compound 156** | 353694 | 10000 | Inactives DUD-E | Low Activity |
| **Compound 157** | 353724 | 10000 | Inactives DUD-E | Low Activity |
| **Compound 158** | 353725 | 10000 | Inactives DUD-E | Low Activity |
| **Compound 159** | 353796 | 10000 | Inactives DUD-E | Low Activity |
| **Compound 160** | 419676 | 10000 | Inactives DUD-E | Low Activity |
| **Compound 161** | 457053 | 10000 | Inactives DUD-E | Low Activity |
| **Compound 162** | 457055 | 10000 | Inactives DUD-E | Low Activity |
| **Compound 163** | 458084 | 10000 | Inactives DUD-E | Low Activity |
| **Compound 164** | 463136 | 10000 | Inactives DUD-E | Low Activity |
| **Compound 165** | 549662 | 10000 | Inactives DUD-E | Low Activity |
| **Compound 166** | 581046 | 10000 | Inactives DUD-E | Low Activity |
| **Compound 167** | 581050 | 10000 | Inactives DUD-E | Low Activity |
| **Compound 168** | 682702 | 10000 | Inactives DUD-E | Low Activity |
| **Compound 169** | 682968 | 10000 | Inactives DUD-E | Low Activity |
| **Compound 170** | 683700 | 10000 | Marginal-Inactives combined DUD-E | Low Activity |
| **Compound 171** | 09F | 11000 | PDB bank | Low Activity |
| **Compound 172** | 384955 | 20000 | Inactives DUD-E | Low Activity |
| **Compound 173** | 384956 | 20000 | Inactives DUD-E | Low Activity |
| **Compound 174** | 415162 | 20000 | Inactives DUD-E | Low Activity |
| **Compound 175** | 419727 | 20000 | Inactives DUD-E | Low Activity |
| **Compound 176** | 419730 | 20000 | Inactives DUD-E | Low Activity |
| **Compound 177** | 419732 | 20000 | Inactives DUD-E | Low Activity |
| **Compound 178** | 419733 | 20000 | Inactives DUD-E | Low Activity |
| **Compound 179** | 615866 | 20000 | Inactives DUD-E | Low Activity |
| **Compound 180** | CHEMBL1214215 | 42460 | Inactives combined DUD-E | Low Activity |
| **Compound 181** | CHEMBL1214158 | 42820 | Inactives combined DUD-E | Low Activity |
| **Compound 182** | CHEMBL1214157 | 44250 | Inactives combined DUD-E | Low Activity |
| **Compound 183** | CHEMBL1214218 | 45250 | Inactives combined DUD-E | Low Activity |
| **Compound 184** | CHEMBL200510 | 100000 | Inactives combined DUD-E | Low Activity |
| **Compound 185** | CHEMBL264296 | 100000 | Inactives combined DUD-E | Low Activity |
| **Compound 186** | CHEMBL374512 | 100000 | Inactives combined DUD-E | Low Activity |
| **Compound 187** | CHEMBL1546 | 100000 | Inactives combined DUD-E | Low Activity |
| **Compound 188** | CHEMBL521824 | 100000 | Inactives combined DUD-E | Low Activity |
| **Compound 189** | CHEMBL493474 | 100000 | Inactives combined DUD-E | Low Activity |
| **Compound 190** | CHEMBL1086647 | 100000 | Inactives combined DUD-E | Low Activity |
| **Compound 191** | CHEMBL1089043 | 100000 | Inactives combined DUD-E | Low Activity |
| **Compound 192** | CHEMBL1092153 | 100000 | Inactives combined DUD-E | Low Activity |
| **Compound 193** | CHEMBL1083529 | 100000 | Inactives combined DUD-E | Low Activity |
| **Compound 194** | CHEMBL1090060 | 100000 | Inactives combined DUD-E | Low Activity |
| **Compound 195** | CHEMBL1203968 | 100000 | Inactives combined DUD-E | Low Activity |
| **Compound 196** | 1W1 | 200000 | PDB bank | Low Activity |
| **Compound 197** | CHEMBL487288 | 250000 | Inactives combined DUD-E | Low Activity |
| **Compound 198** | CHEMBL521384 | 250000 | Inactives combined DUD-E | Low Activity |
| **Compound 199** | CHEMBL487493 | 250000 | Inactives combined DUD-E | Low Activity |
| **Compound 200** | CHEMBL519079 | 250000 | Inactives combined DUD-E | Low Activity |
| **Compound 201** | CHEMBL521590 | 250000 | Inactives combined DUD-E | Low Activity |
| **Compound 202** | CHEMBL490184 | 250000 | Inactives combined DUD-E | Low Activity |
| **Compound 203** | CHEMBL489368 | 250000 | Inactives combined DUD-E | Low Activity |
| **Compound 204** | CHEMBL488104 | 250000 | Inactives combined DUD-E | Low Activity |
| **Compound 205** | CHEMBL256581 | 500000 | Inactives combined DUD-E | Low Activity |
| **Compound 206** | CHEMBL403087 | 500000 | Inactives combined DUD-E | Low Activity |
| **Compound 207** | CHEMBL256162 | 500000 | Inactives combined DUD-E | Low Activity |
| **Compound 208** | CHEMBL467003 | 600000 | Inactives combined DUD-E | Low Activity |
| **Compound 209** | CHEMBL466383 | 600000 | Inactives combined DUD-E | Low Activity |
| **Compound 210** | CHEMBL444170 | 600000 | Inactives combined DUD-E | Low Activity |
| **Compound 211** | CHEMBL1222143 | 1.00E+06 | Inactives combined DUD-E | Low Activity |
| **Compound 212** | CHEMBL1222298 | 1.00E+06 | Inactives combined DUD-E | Low Activity |
| **Compound 213** | CHEMBL1222299 | 1.00E+06 | Inactives combined DUD-E | Low Activity |
| **Compound 214** | CHEMBL512297 | 1.6E+06 | Inactives combined DUD-E | Low Activity |
| **Compound 215** | CHEMBL219680 | 2.00E+06 | Inactives combined DUD-E | Low Activity |

**Table S2.** Statistical analysis of the database used in the study taking into account some key properties of the compounds calculated with Qikprop

|  | | **Molecular Weight** | **(%) Human Oral Absortion** | **Lipinsky’s five rules** | **QPlogBB** | **#Stars** | **QPlogPoct/w** |
| --- | --- | --- | --- | --- | --- | --- | --- |
| **N** |  | 215 | 215 | 215 | 215 | 215 | 215 |
| **Average** | | 498.93 | 77.59 | 0.84 | -1.02 | 1.30 | 3.81 |
| **Standard error of the average** | | 5.928 | 1.133 | 0.048 | 0.055 | 0.123 | 0.105 |
| **Median** | | 516.00 | 77.00 | 1.00 | -0.992 | 1.00 | 3.83 |
| **Modal value** | | 516 | 100 | 1 | -1.75^a^ | 0 | 2.72^a^ |
| **Standard deviation** | | 86.92 | 16.61 | 0.71 | 0.802 | 1.793 | 1.54 |
| **Variance** | | 7554.45 | 275.94 | 0.49 | 0.644 | 3.215 | 2.36 |
| **Mínimum value** | | 114 | 32 | 0 | -3.62 | 0 | -1.63 |
| **Máximum value** | | 701 | 100 | 2 | 0.83 | 10 | 8.64 |


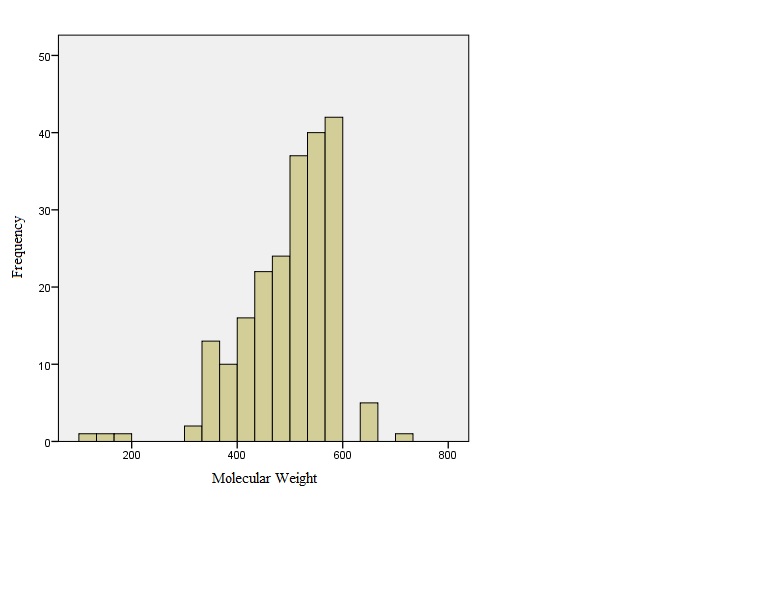


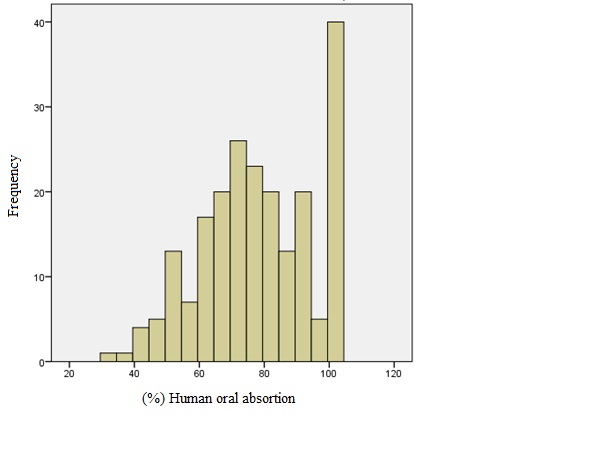


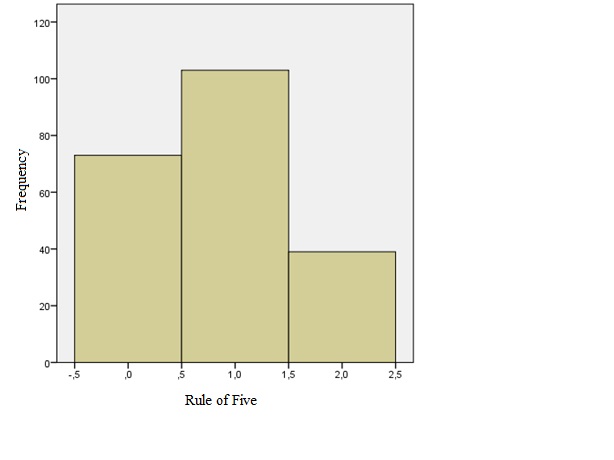


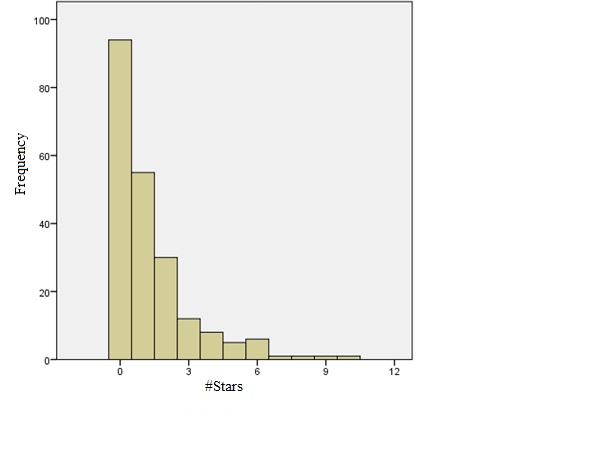


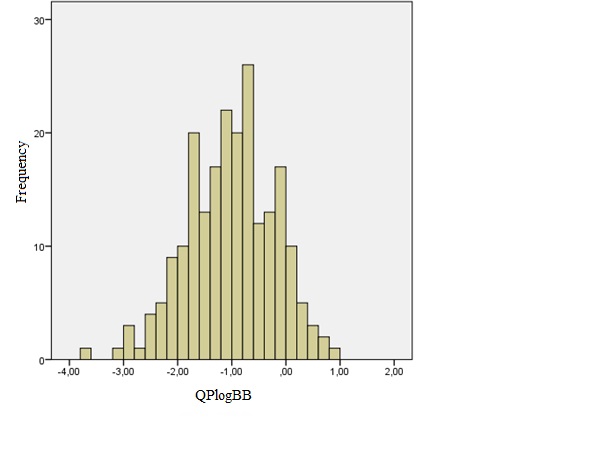


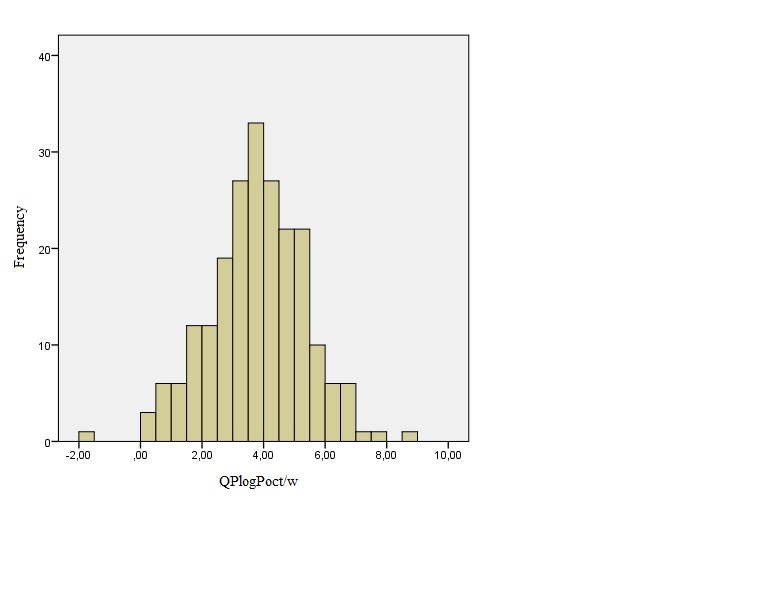


**Figures S1-S6** Histograms including some of the key properties calculated in Qikprop and the frequency of the values.

**Table S3.** Performances of the best QSAR classifiers inferred for HS1 reduced subsets, where the seven-reduced subsets were obtained by removing one molecular descriptor at once. The best model is highlighted in bold.

| Subset | Cardinality | Method | %CC | ROC | Confusion Matrix | |  |
| --- | --- | --- | --- | --- | --- | --- | --- |
| HS1 - | 7 | RF | 77 | 0.85 | *High* | *Low* |  |
| RDF080m |  |  |  |  | 27 | 4 | *High* |
|  |  |  |  |  | 8 | 13 | *Low* |
| HS1 - | 7 | RF | 77 | 0.78 | *High* | *Low* |  |
| GGI7 |  |  |  |  | 28 | 3 | *High* |
|  |  |  |  |  | 9 | 12 | *Low* |
| HS1 - | 7 | RC | 77 | 0.82 | *High* | *Low* |  |
| H1e |  |  |  |  | 25 | 5 | *High* |
|  |  |  |  |  | 7 | 14 | *Low* |
| HS1 - | 7 | RC | 75 | 0.76 | *High* | *Low* |  |
| H6m |  |  |  |  | 25 | 5 | *High* |
|  |  |  |  |  | 8 | 13 | *Low* |
| HS1 - | 7 | NN | 83 | 0.82 | *High* | *Low* |  |
| Mor31p |  |  |  |  | 26 | 5 | *High* |
|  |  |  |  |  | 4 | 17 | *Low* |
| ***HS1 -*** | ***7*** | ***RF*** | ***85*** | ***0.85*** | ***High*** | ***Low*** |  |
| ***MW*** |  |  |  |  | ***28*** | ***3*** | ***High*** |
|  |  |  |  |  | ***5*** | ***16*** | ***Low*** |
| HS1 - | 7 | RC | 79 | 0.80 | *High* | *Low* |  |
| nCrs |  |  |  |  | 27 | 4 | *High* |
|  |  |  |  |  | 7 | 14 | *Low* |
| HS1 - | 7 | RF | 81 | 0.86 | *High* | *Low* |  |
| N-069 |  |  |  |  | 27 | 4 | *High* |
|  |  |  |  |  | 6 | 15 | *Low* |

**Table S4.** Performances of the best QSAR models inferred for HS1 reduced subsets, where the six-reduced subset were obtained by removing one molecular descriptor at once from the best subset of Table 6. The best model is highlighted in bold.

| Subset | Cardinality | Method | %CC | ROC | Confusion Matrix | |  |
| --- | --- | --- | --- | --- | --- | --- | --- |
| ***HS1 - MW -*** | ***6*** | ***RF*** | ***85*** | ***0.88*** | ***High*** | ***Low*** |  |
| ***RDF080m*** |  |  |  |  | ***30*** | ***1*** | ***High*** |
|  |  |  |  |  | ***7*** | ***14*** | ***Low*** |
| HS1 - MW - | 6 | RF | 75 | 0.81 | *High* | *Low* |  |
| GGI7 |  |  |  |  | 25 | 5 | *High* |
|  |  |  |  |  | 8 | 13 | *Low* |
| HS1 - MW - | 6 | RC | 79 | 0.84 | *High* | *Low* |  |
| H1e |  |  |  |  | 27 | 4 | *High* |
|  |  |  |  |  | 7 | 14 | *Low* |
| HS1 - MW - | 6 | RC | 85 | 0.84 | *High* | *Low* |  |
| H6m |  |  |  |  | 27 | 4 | *High* |
|  |  |  |  |  | 4 | 17 | *Low* |
| HS1 - MW - | 6 | NN | 83 | 0.79 | *High* | *Low* |  |
| Mor31p |  |  |  |  | 30 | 1 | *High* |
|  |  |  |  |  | 8 | 13 | *Low* |
| HS1 - MW - | 6 | RC | 79 | 0.80 | *High* | *Low* |  |
| nCrs |  |  |  |  | 27 | 4 | *High* |
|  |  |  |  |  | 6 | 15 | *Low* |
| HS1 - MW - | 6 | RC | 73 | 0.82 | *High* | *Low* |  |
| N-069 |  |  |  |  | 24 | 7 | *High* |
|  |  |  |  |  | 7 | 14 | *Low* |

**Table S5.** Performances of the best QSAR classifiers inferred for HS1 reduced subsets, where the five reduced subset were obtained by removing one molecular descriptor at once from the best subset of Table 7.

| Subset | Cardinality | Method | %CC | ROC | Confusion Matrix | |  |
| --- | --- | --- | --- | --- | --- | --- | --- |
| HS1 - MW - | 5 | RF | 83 | 0.85 | *High* | *Low* |  |
| RDF080m - GGI7 |  |  |  |  | 27 | 4 | *High* |
|  |  |  |  |  | 5 | 16 | *Low* |
| HS1 - MW - | 5 | RC | 75 | 0.83 | *High* | *Low* |  |
| RDF080m - H1e |  |  |  |  | 24 | 7 | *High* |
|  |  |  |  |  | 6 | 15 | *Low* |
| HS1 - MW - | 5 | RF | 83 | 0.85 | *High* | *Low* |  |
| RDF080m - H6m |  |  |  |  | 29 | 2 | *High* |
|  |  |  |  |  | 7 | 14 | *Low* |
| HS1 - MW - | 5 | RF | 79 | 0.79 | *High* | *Low* |  |
| RDF080m - Mor31p |  |  |  |  | 28 | 3 | *High* |
|  |  |  |  |  | 8 | 13 | *Low* |
| HS1 - MW - | 5 | RF | 75 | 0.83 | *High* | *Low* |  |
| RDF080m - nCrs |  |  |  |  | 25 | 6 | *High* |
|  |  |  |  |  | 7 | 14 | *Low* |
| HS1 - MW - | 5 | RF | 83 | 0.89 | *High* | *Low* |  |
| RDF080m - N-069 |  |  |  |  | 29 | 2 | *High* |
|  |  |  |  |  | 7 | 14 | *Low* |
